# Alternative Options for Complex, Recurrent Pain States Using Cannabinoids, Psilocybin, and Ketamine: A Narrative Review of Clinical Evidence

**DOI:** 10.3390/neurolint14020035

**Published:** 2022-05-18

**Authors:** Amber N. Edinoff, Juliana M. Fort, Christina Singh, Sarah E. Wagner, Jessica R. Rodriguez, Catherine A. Johnson, Elyse M. Cornett, Kevin S. Murnane, Adam M. Kaye, Alan D. Kaye

**Affiliations:** 1 Department of Psychiatry and Behavioral Medicine, Louisiana State University Health Science Center Shreveport, Shreveport, LA 71103, USA; juliana.fort@lsuhs.edu (J.M.F.); christina.singh@lsuhs.edu (C.S.); kevin.murane@lsuhs.edu (K.S.M.); 2 School of Medicine, Louisiana State University Health Science Center Shreveport, Shreveport, LA 71103, USA; sew001@lsuhs.edu (S.E.W.); jrr001@lsuhs.edu (J.R.R.); caj001@lsuhs.edu (C.A.J.); 3 Department of Anesthesiology, Louisiana State University Health Science Center Shreveport, Shreveport, LA 71103, USA; elyse.bradley@lsuhs.edu (E.M.C.); alan.kaye@lsuhs.edu (A.D.K.); 4 Department of Pharmacology, Louisiana State University Health Science Center Shreveport, Toxicology & Neuroscience, Shreveport, LA 71103, USA; 5 Louisiana Addiction Research Center, Shreveport, LA 71103, USA; 6 Department of Pharmacy Practice, Thomas J. Long School of Pharmacy and Health Sciences, University of the Pacific, Stockton, CA 95211, USA; akaye@pacific.edu

**Keywords:** plant-based medications, alternative pain medications, chronic pain, opioids, cannabinoids, psilocybin

## Abstract

With emerging information about the potential for morbidity and reduced life expectancy with long-term use of opioids, it is logical to evaluate nonopioid analgesic treatments to manage pain states. Combinations of drugs can provide additive and/or synergistic effects that can benefit the management of pain states. In this regard, tetrahydrocannabinol (THC) and cannabidiol (CBD) modulate nociceptive signals and have been studied for chronic pain treatment. Psilocybin, commonly known as “magic mushrooms”, works at the serotonin receptor, 5-HT_2A_. Psilocybin has been found in current studies to help with migraines since it has a tryptamine structure and works similarly to triptans. Psilocybin also has the potential for use in chronic pain treatment. However, the studies that have looked at alternative plant-based medications such as THC, CBD, and psilocybin have been small in terms of their sample size and may not consider the demographic or genetic differences in the population because of their small sample sizes. At present, it is unclear whether the effects reported in these studies translate to the general population or even are significant. In summary, additional studies are warranted to evaluate chronic pain management with alternative and combinations of medications in the treatment of chronic pain.

## 1. Introduction

The International Association for Study of Pain defines it as “an unpleasant sensory and emotional experience associated with or resembling that associated with, actual or potential tissue damage” [1]. Many patients experience acute pain postoperatively, and state laws have recently emerged to limit opioid prescriptions to between 3 and 7 days though pain may last longer. Acute pain is different from chronic pain and is described as an unpleasant response to tissue trauma, usually self-limiting. It serves a protective role in allowing time for healing by minimizing harmful behaviors. Acute post-operative pain typically lasts up to ten days after surgery [2]. Chronic pain, however, lasts beyond the time needed for the injured tissue to heal and may last indefinitely [2].

The prevalence of chronic post-surgical pain (CPSP) is about 10% in all surgeries. CPSP is pain at the incision site that persists longer than a month after it should take for most injured tissues to heal. The onset is usually between 3 and 6 months after surgery [3]. In addition, it is not uncommon for nerves to be iatrogenically damaged during surgical procedures, causing persistent pain post-operatively [2]. This type of pain is difficult to treat due to general unresponsiveness to analgesics [4].

Since both peripheral and central sensitization seem to be involved in the development of CPSP, these mechanisms may yield novel drug targets. Tetrahydrocannabinol (THC) and cannabidiol (CBD) modulate nociceptive signals and can be considered a possible treatment for CPSP. Synergistic pain relief with low doses of THC and CBD has also been reported. Therefore, combination treatment may be considered to prevent acute post-operative pain in patients with a risk of nerve damage [3]. Psilocybin, commonly known as “magic mushrooms”, works at the serotonin receptor, 5-HT_2A_. Psilocybin has been found in current studies to help with migraines since it has a tryptamine structure and works similarly to triptans. Psilocybin has the potential for use in chronic pain treatment as well.

Several other factors contribute to the pain syndrome that must not be underestimated. Stress and the environment may contribute to pain intensity and persistence. In addition, pre-existing chronic pain before surgery and anxiety afterward may increase the risk of pain persistence [2]. Since several factors influence chronic pain development, a single treatment may not be effective for most patients [3]. This must be taken into consideration before prescribing medications for pain management.

## 2. Current Treatment of Chronic Pain

### 2.1. The Ladder of Treatment for Chronic Pain

The World Health Organization (WHO) has developed an analgesic ladder with several steps to guide healthcare providers in treating patients with chronic pain [5]. The first step is using nonopioid analgesics such as NSAIDs, which reduce inflammation by inhibiting COX-1 and COX-2. COX-2 is chiefly responsible for inflammation and is thus the target of NSAIDs in pain relief. These must be prescribed with caution in large doses due to side effects, such as gastrointestinal ulcers. Selective COX-2 inhibitors such as celecoxib reduce the risk of the adverse effect of ulcers but may lead to increased incidence of thrombotic cardiovascular events [5].

The second step is using weak opioids, such as codeine, tramadol, and dihydrocodeine. These medications reduce chemicals that activate opioid receptors in the CNS and reduce the transmission of nociceptive signals. It should be noted that even “weak” opioids have the same side effect profile as more potent opioids and have not been shown to have a decreased risk of addiction when compared to their more potent counterparts [6].

The third step is the use of stronger opioids, such as hydrocodone. They activate G-protein-coupled opioid receptors, which promote K^+^ entry and inhibit Ca^2+^ entry into the nerve cell. The side effect profile is more pronounced than in weak opioids. Respiratory depression, for instance, can be fatal even at therapeutic doses. Care must be taken to ensure the patient begins on a low dose. The higher the dose, the better the pain control, so the dose can be increased to combat tolerance and improve pain control. However, the side effects become more severe at higher doses. Reviews have shown short-term efficacy in musculoskeletal and neuropathic pain conditions, but use for periods lasting longer than six months is not recommended [5].

### 2.2. Side Effects of Opioids

Opioids are effective in the treatment of chronic pain; however, they are also associated with numerous side effects. There was an increased risk of experiencing adverse events with opioids than with placebo (relative risk = 1.42). These adverse events included constipation, dizziness, drowsiness, fatigue, hot flushes, diaphoresis, nausea, vomiting, and pruritis. The study also demonstrated a 42% higher risk of adverse events and 175% increased risk of serious adverse events associated with opioids when compared to placebo [7]. Due to selective pain sensitization, long-term opioid use is also linked to opioid-induced hyperalgesia. This implies that opioids may intensify central sensitization (CS) by activating pronociceptive pathways [8]. CS is discussed in the next section.

Opioids have been shown to alter sleep regulation leading to sleep apnea and poor sleep quality. Furthermore, respiratory depression is associated with sleep-disordered breathing and may result in death since this can happen in opioid use. These agents have also been linked to depressive and anxiety disorders as well as sexual dysfunction. In addition, there is an increased risk of addiction to opioids with increasing dosages. Related to the side effect profile and risk of addiction and death, long-term use of opioids may only be recommended for a small subset of people living with chronic non-cancer pain [7]. This leads to a gap in the treatment of chronic pain if opioids are left out of the equation.

### 2.3. Central Sensitization Treatments

CS is a pain mechanism that amplifies signaling within the central nervous system, leading to pain hypersensitivity. This mechanism contributes to an augmented response to several types of stimuli such as mechanical pressure, chemicals, sound, and temperature. Drugs such as acetaminophen act centrally and thus may be used to treat CS pain in diseases, e.g., fibromyalgia, which is due to central pain processing abnormalities. SNRIs such as duloxetine have also shown promise in treating fibromyalgia and osteoarthritis. This may be due to a resulting decrease in CNS hyperexcitability; however, the actual mechanism is unclear [8].

Several other drug classes directly target the central nervous system and are recommended to treat CS pain. A new class of drugs known as the mu-opioid receptor agonist and norepinephrine reuptake inhibitor (MOR-NRI) has analgesic effects explained by a synergistic interaction between stimulation of the MOR and inhibition arising from norepinephrine reuptake inhibition. N-methyl-D-aspartate (NMDA) receptor antagonists have demonstrated effective analgesia by blocking neuronal excitation produced by NMDA receptor stimulation, which may block hyperalgesia. GABA agonists such as pregabalin work by binding to Ca^2+^ channels and blocking Ca^2+^ influx during depolarization, resulting in a reduction in glutamate, norepinephrine, and substance P release [8].

### 2.4. Multimodal Treatments

Another consideration in the treatment of chronic pain is combination drug therapy. This combination of drug therapies is known as multimodal treatments. Because pain has several mechanisms, prescribing different drugs that target each of those mechanisms is a possible strategy. Most pharmacologic agents alone provide a positive response in some people but since pain can be caused by many different mechanisms, a single agent may not completely target the cause of the pain or only partially relieve the pain. Therefore, combinations of drugs provide additive and/or synergistic effects to improve pain by targeting different mechanisms [9].

Combination drug therapy shows promise for numerous pain conditions, particularly neuropathic pain. For example, when gabapentenoids and tricyclic antidepressants are used in combination, they are more efficacious in the treatment of diabetic peripheral neuropathy and postherpetic neuralgia than with either drug alone [9]. Chronic low back pain is another condition that may benefit from combination drug therapy due to the presence of both neuropathic and nociceptive pain mechanisms. The Multimodal treatment model of chronic pain treatment also includes non-pharmacologic interventions as well. This can include both physical and occupational therapy. It can also include psychological interventions such as cognitive-behavioral therapy, yoga, or tai chi [10]. The addition of these treatments help address other factors that may be making the pain be perceived as more painful due to psychological factors such as depression or stress.

## 3. Alternative Medications

As the search for adequate chronic pain management continues, neuroactive substances such as some plant-based past drugs of abuse have been called into question as possible treatments for chronic pain. These drugs had previously been stigmatized for their abusive properties that cause hallucinogenic episodes. However, as the field of neuroplasticity has expanded, recent studies on alternative, plant-based drugs have revealed possible psychoplasmic capabilities that can lead to relief from chronic pain.

Compounds such as lysergic acid diethylamide (LSD) and 4-phosphorloxy-N, N-dimethyltryptamine (psilocybin) are serotonergic hallucinogens that are beginning to emerge as a promising target. These chemical compounds have been known to act on the serotonergic 5-HT_2A_ receptors. They play a role in learning, memory, hallucinations, and neurocognitive disorders. These activate many pathways which release oxytocin and acetylcholine [11]. Further investigation carried out by Ly et al. found that the neuronal remodeling is seen in these drugs through the activation of the 5-HT_2A_, mTOR, and TrkB (tropomyosin receptor kinase B) receptors. mTOR activation is an important regulator of protein synthesis needed for neuronal growth [12,13]. TrkB receptors are linked to learning through long-term memory formation and plasticity in the hippocampus [14].

Alternative drugs, which have been used for other means, promise neuronal synaptic modulators. Ketamine is used for sedation maintenance and causes dissociative anesthesia. Ketamine is currently thought to preferentially block inhibitory GABAergic interneuron at the N-methyl-D-aspartate receptor (NMDAR). This blockage increases extracellular glutamate levels in the prefrontal cortex. It activates the release of BDNF (brain-derived neurotrophic factor), elongation factor 2 (eEF2), mTOR, and Glycogen synthase kinase-3 (GSK-3), which are key regulators of growth in the GABAergic neurons [15].

When looking at these electrophysiological changes in the brain, research groups have found that this drug administration causes broadband desynchronization and a disconnect in neurons in the sensorimotor cortex on EEG monitoring [16]. The results are an overall resetting due to decreased global activity and connectivity. This could perhaps lead to reset in the perception of pain or lead to a decrease in neuropathic pain.

### Cannabidiol as a Possible Treatment

Related to the recent legalization of cannabis in several states in the United States, there has been growing interest in alternative therapies for chronic pain, including tetrahydrocannabinol (THC) and cannabidiol (CBD). Both THC and CBD come from the plant *Cannabis sativa*, with THC being the psychoactive component and CBD the non-psychoactive component. They both act on endocannabinoid receptors located in the brain [17]. THC has been associated with the high sensation that a person feels when consuming cannabis [18]. THC has also been associated with psychosis in some users. Studies suggest that smoking high potency marijuana (high in THC) every day could increase the chances of developing psychosis by nearly five times compared to those who have never used marijuana [19]. Research has suggested that CBD may have analgesic, anti-inflammatory, anticonvulsant, muscle relaxant, anxiolytic, and even antipsychotic activity [20].

## 4. Mechanisms of Action

Although the mechanism by which these mild-altering drugs exert their analgesic effects is not fully understood, their mind-altering qualities have been attributed to serotonin 2A receptor agonism, specifically 5-HT_2A_ [21]. This is thought to alter brain regions’ functional connectivity (FC) which also plays a role in pain perception. Normally, descending inhibitory 5-HT pathways in the spinal cord regulate the transmission of pain signals by inhibiting c-fiber responses in the dorsal horn. If this process goes awry, it could lead to hyperalgesia. Rat models have shown that after nerve ligation, only activation of the 5-HT_2A_ receptor resulted in persistent 5-HT descending inhibition, suggesting the role of 5-HT_2A_ receptors involved in nerve injury pain [22].

5-HT_2A_ receptors in the dorsal root ganglia (DRG) have enhanced inflammatory pain, and 5-HT_2A_ antagonists then reduce pain responses to inflammatory stimuli. Rats and mice treated with 2′,3′-dideoxycytidine (ddC) exhibit thermal allodynia and mechanical hypersensitivity. The selective 5-HT_2A_ antagonist, glemanserin, can reverse this. This effect is not seen in 5-HT_2A_ receptor knockout mice. This is likely due to increased 5-HT_2A_ expression in the DRG from ddC, sensitizing spinal pain responses [22].

Studies have also shown that these drugs alter connectivity patterns within the brain. In analyzing fMRI studies involving psilocybin, Carhart-Harris et al. proposed that they “disintegrate” brain networks and increase the connectivity established within networks. When combined with psychotherapy, these changes can potentially treat numerous psychiatric conditions, such as depression and anxiety. Although the mechanisms for chronic pain sensation are not completely understood, distraction and changes in mood can affect the perception of pain. Dysphoric mood states increase the perception of chronic pain. This suggests that these alternative drugs can indirectly treat chronic pain by affecting the individual’s perception of pain [22].

Activating the 5-HT_2A_ receptor upregulates genes associated with neuroplasticity and suppresses TNF-a-induced inflammation. These alternative drugs downregulate 5-HT_2A_ receptor binding sites, likely due to redistribution of the receptor intracellularly from the cell surface. Although there have not been many studies to demonstrate this effect in DRG, it could potentially counteract the sensitization of nociceptive responses found in neuropathic pain [22].

## 5. Clinical Studies

### 5.1. Chronic Pain and Cannabinoids

A randomized, double-blinded, crossover study evaluated the therapeutic window of inhaled Δ^9^-tetrahydrocannabinol, another formulation of THC, in 27 chronic pain patients [23]. Using the Syqe Inhaler on three occasions, patients received a placebo, 0.5 mg THC, or 1.0 mg THC. After inhaling 0.5 mg THC, average plasma THC concentration (C_max_) was 14.3 ± 7.7 ng/mL and time to peak THC concentration (T_max_) was 3.7 ± 1.4 min. With 1.0 mg of THC, a C_max_ of 33.8 ± 25.7 ng/mL and a T_max_ of 4.4 ± 2.1 min were yielded. The visual analog scale (VAS) pain scores following 1.0 mg THC demonstrated greater pain reduction compared to the placebo and 0.5 mg THC (*p* = 0.0015 and *p* = 0.0058, respectively). VAS pain scores declined by 39% and 25% in the 1.0 mg THC group and 0.5 mg THC group, respectively. A 2 point pain score reduction was reported by 70% of 1.0 mg THC patients, 64% of 0.5 mg THC patients, and 26% of placebo patients [23]. Mild adverse events, the most common being drug high (20%), peaked within 30 min of inhalation. Events occurred in all groups but more frequently with THC (placebo = 41, 0.5 mg THC = 60, 1.0 mg THC = 66). The authors concluded that low, precise doses of inhaled THC demonstrated dose-dependent pharmacokinetics and provided significant analgesia to patients with chronic pain, but long-term effects were undetermined [23].

Long-term cannabis safety was assessed in a 1-year prospective cohort study on Canadians with non-cancer-related chronic pain [24]. Two hundred fifteen patients received herbal cannabis (12.5 ± 1.5% THC) and reported a median dose of 2.5 g/d. The 216 control patients did not receive cannabis. Both groups experienced serious adverse events, yet the risk was not higher in cannabis users (IRR = 0.82, 95% CI = 0.46–1.46). However, medical cannabis users exhibited a greater risk of non-serious adverse events (IRR = 1.64, 95% CI = 1.35–1.99) [24]. Cannabis increased risk of neurological (IRR = 2.05, 95% CI = 1.46–2.86), pulmonary (IRR = 1.77, 95% CI = 1.16–2.70), infectious (IRR = 1.51, 95% CI = 1.04–2.20), and psychiatric (IRR = 2.74, 95% CI = 1.45–5.18) effects. Authors conclude that adverse events in cannabis patients were mild to moderate, but 2.5 g/d may be safe for experienced cannabis users with chronic pain. Because 66% of patients were experienced users, new users could not evaluate safety accurately [24].

A randomized, double-blinded, placebo-controlled study assessed the effects of THC on chronic abdominal pain. Sixty-two patients with chronic pancreatitis-related abdominal pain or post-surgical abdominal pain were randomized into a treatment group (standardized THC tablets) or control group (identical placebo tablets). For the first five days, 3 mg tablets were taken three times a day, and 5 mg tablets were taken three times a day for the following five days. During days 11 through 52, patients maintained a constant tablet dose of 8 mg three times a day. Post-treatment evaluation of mean VAS pain scores revealed no significant benefit of THC compared to the placebo (placebo group = 37% reduction, THC group = 40% reduction, *p* = 0.901) [25].

A randomized, double-blinded, placebo-controlled trial observed the clinical effects of adjunctive oral cannabis spray on 397 advanced cancer patients with pain refractory to opioids [26]. Either nabiximol in a 1:1 ratio of Δ^9^-tetrahydrocannabinol and cannabidiol (THC: CBD) or placebo was randomly assigned. Patients self-administered single spray doses for two weeks and added one spray each day, and a tolerable dose was maintained for three weeks. Overall, nabiximol was not superior to the placebo in analgesia based on numerical rating scale (NRS) scores (10.7% improvement vs. 4.5% improvement, *p* = 0.085) [26].

Another randomized, double-blinded clinical trial studied adjunctive dronabinol, a synthetic THC, on 30 patients taking opioids for chronic non-cancer pain [27]. In phase 1, patients received capsules of a placebo, 10 mg dronabinol, or 20 mg dronabinol at three visits. Total pain relief (0 = no relief, 10 = total relief) was summed on a scale of 1 to 10. The results showed that dronabinol provided significant analgesia compared to the placebo (20 mg = 41.7, *p* < 0.01; 10 mg = 39.7, *p* < 0.05). Dry mouth, fatigue, and drowsiness, the most common adverse events, occurred more often with 20 mg dronabinol compared to the placebo (*p* < 0.0001, *p* < 0.0001, and *p* < 0.05, respectively). Patients in both dronabinol groups reported greater pain relief on the 0 to 10 pain relief scale compared to the placebo group (10 mg = 5.93, 20 mg = 5.93, *p* < 0.01 for both). In phase 2 of this study, patients were treated with open-label dronabinol for four weeks. They reported a significant decrease from baseline average pain scores (*p* < 0.001), but a comparison of consecutive weeks did not reveal a difference. The study concludes that dronabinol might be a useful adjunctive therapy for chronic non-cancer pain [27].

The effect of various oral spray cannabis preparations on chronic neuropathic pain was explored in a randomized, double-blinded, crossover trial [28]. All 34 patients received an open-label THC:CBD mixture for two weeks and tracked their most severe symptoms (S1 and S2). Patients subsequently received a random cannabis extract each week for 8 weeks: 2.5 mg THC, 2.5 mg CBD, 2.5 mg THC + 2.5 mg CBD (THC:CBD), or placebo. Median cumulative S1 VAS scores were 5.9, 5.45, 4.63, and 4.4 for the placebo, CBD, THC, and THC:CBD groups, respectively (*p* < 0.001). S2 VAS scores were 4.98, 5.03, 4.08, and 4.28 for the placebo, CBD, THC, and THC:CBD groups, respectively (*p* < 0.001). S1 scores significantly improved with THC (*p* < 0.01) and THC:CBD (*p* < 0.05) compared to the placebo, and S2 scores significantly improved with THC (*p* < 0.001). An improved sleep quality was reported in the THC: CBD (*p* < 0.001), THC (*p* < 0.001), and CBD (*p* < 0.05) groups compared to the placebo group. Authors concluded that THC and THC:CBD extracts provided the greatest improvements in pain and sleep quality [28]. Table 1 summarizes the studies discussed here regarding cannabinoids and chronic pain.

### 5.2. Neuropathic Pain and Cannabinoids

Another randomized, double-blinded, crossover study analyzed the efficacy of herbal cannabis in neuropathic pain [29]. Twenty-three patients with post-surgical or post-traumatic neuropathic pain were randomly assigned varying THC potencies: 0%, 2.5%, 6%, or 9.4%. Single 25 mg doses were inhaled via titanium pipe three times a day for five days with a 9-day washout before each cross-over. Average daily VAS pain scores for the 9.4% THC group were significantly reduced compared to the placebo (9.4% THC = 5.4, 0% THC = 6.1, *p* = 0.023). Based on the Leeds Sleep Evaluation Questionnaire and EQ-5D assessment, 9.4% THC improved sleep quality and symptoms of anxiety and depression compared to the placebo as well (*p* < 0.05 for both) [29,30,31].

A randomized, double-blinded, placebo-controlled trial studied sublingual THC effects on brain connectivity in 15 male patients with chronic lumbar radicular neuropathic pain. Patients received THC oil (0.2 mg/kg) or placebo oil during two sessions. fMRI scans, used to analyze the connectivity of the anterior cingulate cortex (ACC), located three sensorimotor clusters: right and left secondary somatosensory cortex (SII) and right motor cortex (MI). There was no correlation between ACC connectivity and analgesic response in the placebo or pre-treatment THC groups. However, post-THC treatment revealed a decrease in ACC and sensorimotor cortex functional connectivity in right SII, left SII, and right MI, which correlated with improved pain (r = 0.68, *p* = 0.005; r = 0.66, *p* = 0.007; r = 0.8, *p* = 0.0003, respectively). The study concludes that THC may reduce subjective neuropathic pain by interfering with neural pathways in the ACC [32].

### 5.3. Fibromyalgia and Cannabinoids

Fibromyalgia is a chronic disease, predominantly affecting females, that causes diffuse musculoskeletal pain, altered mood and sleep, and intense fatigue [33]. A double-blinded, crossover study analyzed analgesic responses to inhaled cannabis in fibromyalgia patients. Twenty patients were randomly divided into groups: bedrocan (22% THC, <1% CBD), bediol (6.3% THC, 8% CBD), bedrolite (<1% THC, 9% CBD), or placebo. Vaporized cannabis or placebo was administered on four occasions with a 2-week washout between treatments [34]. Spontaneous pain NRS scores were not significantly different between groups, but 18 patients receiving bediol reported a 30% reduction in spontaneous pain (*p* = 0.01) [34]. Using an algometer to quantify pressure tolerance over the adductor pollicis muscle, bediol, and bedrocan enhanced the pressure threshold (bediol: increase from 9 to 11 kgf, *p* < 0.001; bedrocan: increase from 7 to 9 kgf, *p* = 0.006) [34,35]. The electrical pain test, assessed using two electrodes superior to the right medial malleolus, showed no effect of bedrocan or bediol electrical pain threshold [34,36]. The Bowdle questionnaire, which measures the mind-altering effects or self-perceived “high”, indicated that bedrolite provided a weaker drug high compared to bedrocan (*p* = 0.003) and bediol (*p* < 0.001) [34,37]. Overall, active treatment groups did not significantly reduce spontaneous pain or electrical pain threshold, but THC-containing groups (bediol and bedrocan) improved the pressure pain tolerance [34].

The effects of cannabis on quality of life in fibromyalgia patients were observed in a randomized, double-blinded 8-week clinical trial [38]. Seventeen Brazilian women were randomly placed into a treatment group receiving cannabis oil (24.44 mg/mL THC and 0.51 mg/mL CBD) or a placebo group receiving olive oil. Both groups initially used one drop per day, and doses were adjusted throughout the study. The mean Fibromyalgia Impact Questionnaire (FIQ) scores showed no differences pre-treatment, but a significant decline was noted in the cannabis group post-treatment (cannabis = 30.50 ± 16.18, placebo = 61.22 + 17.30, *p* = 0.005) [38,39]. Another randomized crossover trial compared outcomes of nabilone, a man-made cannabis-based medication, vs. amitriptyline therapy on 32 fibromyalgia patients with chronic insomnia. Patients were randomly assigned identical capsules of 0.5 mg nabilone or 10 mg amitriptyline for two weeks. An identical crossover study occurred following a washout period [40]. Nabilone was superior in sleep quality to amitriptyline according to the Insomnia Severity Index (difference = −3.25, 95% CI = −5.26 to −1.24) [40,41]. The Leeds Sleep Evaluation Questionnaire revealed no differences between groups, but patients taking nabilone experienced more restful sleep (difference = 0.48, 95% CI = 0.01–0.95) and quicker sleep onset (difference = −0.7, 95% CI = −1.36–0.03) [30,40]. Differences in pain, mood and quality of life were insignificant between amitriptyline and nabilone [40].

### 5.4. Multiple Sclerosis and Cannabinoids

Multiple sclerosis (MS) is an inflammatory neurological disorder associated with distressing muscular spasticity in up to 75% of patients and chronic neuropathic pain in 86% of patients [42,43]. A two-phase trial investigated outcomes of ECP002A (Namisol^®^), a THC formulation, on spasticity and pain in 24 patients with MS [44]. During phase 1, patients attended two visits and received three increasing tablet doses of ECP002A or placebo for a 100 min interval. During the four-week phase 2, patients received either a placebo or ECP002A tablet three times a day. Tablets contained 8 mg or 5 mg depending on tolerance in phase 1, and doses were increased by 4.5 mg midway through the study period [44]. Objective spasticity was measured by electrically stimulating the popliteal nerve and recording the ratio of the Hoffmann reflex to the M response (H/M ratio) [45,46]. Both phases found no differences between groups for H/M ratio, NRS for spasticity, or NRS for pain. Post hoc analysis was performed for patients with evidence of pain (*n* = 17) or spasticity (*n* = 18) before phase 2 and revealed a significant decline in NRS pain scores in patients using ECP002A (difference = −1.51, *p* = 0.0198). Regarding spasticity, a significant reduction in NRS was reported following week 2 (difference = −1.23, *p* = 0.0387), but no overall changes in spasticity scores were noted. The study concludes that ECP002A affects spasticity and pain similar to other THC formulas, but higher neurological pathways may influence spasticity in MS [44].

A cohort study observed the effects of THC-CBD (1:1 ratio) oral spray on neurological outcomes in patients with chronic MS. The treatment group consisted of 25 Italian MS patients, and the control group consisted of 14 healthy individuals with similar demographic characteristics. Cannabis dosage increased by one spray per day for 14 days and was maintained for four weeks [47]. A significant improvement from baseline spasticity was noted in MS patients based on the Modified Ashworth Scale to gauge muscle tone, 9-Hole Peg Test to measure fine motor skills, and subjective NRS for spasticity (*p* = 0.001, *p* = 0.018, and *p* = 0.001, respectively) [47,48,49]. VAS pain scores of MS patients were also significantly reduced compared to baseline pain (*p* = 0.005) [47]. Alternatively, the Timed 25-Foot Walk, a gait assessment, did not reveal improvements in MS patients from baseline (*p* = 0.6) [47,50]. Both groups were neurologically assessed using the H/M ratio after electrically stimulating the tibial nerve and cutaneous silent period (CSP), an inhibitory reflex mediated by A-δ fibers in response to transient painful stimuli [51,52]. The H/M ratio showed no changes in MS patients. Before treatment, there was no significant difference between groups in the H/M ratio, CSP duration, or CSP latency. CSP duration in MS patients was significantly prolonged compared to baseline (47.9 ± 6.2, *p* = 0.001), but no associations were found between CSP duration and pain, spasticity, sprays per day, or plasma cannabis levels [47]. Table 2 describes the use of cannabinoids on neuropathic or pain or pain related to chronic pain diseases such as fibromyalgia or MS.

### 5.5. Psilocybin and Chronic Pain

To look at the possible side effects of psilocybin, a study performed in 2012 aimed to investigate the drug’s possible adverse effects in healthy volunteers [53]. This was a double-blind, crossover study that used a range of doses (0, 5, 10, 20, and 30 mg) in 18 healthy volunteers. Of those 18, 4 reported a history of headaches at screening. These doses were given in five 8 h long drug sessions conducted at one-month intervals. Psilocybin doses were increased sequentially in nine randomly selected participants, and the dose was decreased sequentially in the other nine participants. A single placebo session was intermixed with the four active sessions. All reported headaches were mild or moderate and occurred at a mean of 7.0 h after drug administration. The direction of the headache and the percentage of participants who took medicine for their headache occurred in a dose-dependent manner [53]. The authors concluded that psilocybin causes headaches in a dose-dependent manner.

A case report has described a patient who had a leg amputation and then suffered from phantom leg pain [54]. The pain for the patient was intractable in nature. This patient was given psilocybin paired with mirror visual feedback (MVF). The patient described the pain as a nail boring into their leg. During this MVF, a “telescoping” nail was used where the authors had created the illusion of a nail being removed with corresponding pain relief. This pairing of psilocybin and MVF produced a synergistic effect, which led to the complete elimination of the phantom limb pain and a reduction in paroxysmal episodes [54]. Table 3 summarizes the studies discussed in this section regarding psilocybin.

## 6. Conclusions

Chronic pain management is a complex problem across healthcare and is a significant factor in the quality of life for affected individuals. At present, current pain treatments, such as opioids, are limited by desensitization, side effects, tolerance, and dependence. This has led researchers to look for strategies to manage pain through other mechanisms, combinations of drugs, and alternative therapies. Studies on THC and CBD have been performed. They have shown some efficacy in treating certain chronic pain diseases such as multiple sclerosis and fibromyalgia, thus decreasing the severity of pain in these patients. These studies, however, have been small in terms of their sample size and may not take into account demographic or genetic differences in the population because of their small sample sizes. It is unclear if the effects reported in these studies would translate to the general population or even are significant, necessitating larger carefully designed clinical and basic science investigations. The studies regarding psilocybin for pain control have been greatly limited in terms of numbers so no clear conclusion can be drawn at this time. Cannabinoids have some mixed results in chronic pain patients, but most studies reviewed here have shown some positive effects on pain reduction with the most reported adverse effect being the subjective feeling of being “high”. More studies would need to be conducted to thoroughly assess its effectiveness in the general population of those who suffer from chronic pain as well as its long-term safety.

Collectively, these studies have led to the hypothesis that nonopioids with varied mechanisms can provide significant relief in individuals who suffer from chronic pain and associated symptoms such as sleep disorders. New research has shown that these compounds increase neuroplasticity in the nervous system causing disassociation of the synapses for the brain to rewire previous pathways. This is encouraging since the mechanism of chronic pain is currently believed to center around a maladapted neuronal connection that is constitutively active. Using results from studies with THC on chronic pain, fibromyalgia, and multiple sclerosis, there is a new framework and different thought process for considering many different psychoactive compounds.

In summary, studies on THC have shown some modulation of brain activity resulting in improved outcomes. A case study found in the literature regarding psilocybin did illustrate the potential of this drug to be an adjunct in the treatment of pain. More studies on the effects of these potent brain activity modulators, such as psilocybin or LSD, could provide valuable insight into novel strategies for chronic pain treatment. The possible neuroplastic capabilities of these alternative drugs are also promising in treating multiple sclerosis. However, no firm conclusions can be drawn at this time as studies are limited in terms of their size and there are no data on the long-term safety of these drugs. Further studies will need to be performed and are warranted to evaluate the management of chronic pain with alternative and combination medications.

## Figures and Tables

**Table 1:** Studies on chronic pain and cannabinoids.

Author (y)	Groups Studied and Interventions	Results and Findings	Conclusions
Almog et al. (2020) [23]	Adults with chronic pain (VAS ≥ 6) and a medical cannabis license. Exclusion criteria included severe comorbidities, substance abuse, pregnancy, breastfeeding, insufficient contraception.	Dose-dependent pharmacokinetics. Significant VAS pain reduction with 1.0 mg THC (39%, *p* = 0.0015) and 0.5 mg THC (25%, *p* = 0.0058). Two point reduction in 70% of 1.0 mg THC, 63% 0.5 mg THC, and 26% of placebo.	Low, precise doses of inhaled THC in chronic pain patients provided significant analgesia.
Ware et al. (2015) [24]	Adults with chronic non-cancer pain for ≥ 6 months. Exclusion criteria included psychosis history, pregnancy, breastfeeding, unstable cardiac or respiratory disease.	Higher risk of non-serious adverse events with medical cannabis (IRR = 1.64, 95% CI = 1.35–1.99), but no increased risk of serious events.	Mild adverse events occurred with cannabis, but 2.5 g/d may be safe for experienced users with chronic pain.
de Vries et al. (2017) [25]	Adults with abdominal pain (≥3 months, NRS ≥ 3) from chronic pancreatitis or surgery. Exclusion criteria: daily cannabis, previous cannabis sensitivity, severe comorbidity, positive urine drug or alcohol screen, BMI > 36 kg/m^2^, pregnancy, breastfeeding.	No significant difference in VAS pain score reduction between groups (placebo = 37% reduction, THC = 40% reduction, *p* = 0.901).	THC tablets did not provide pain relief for patients with chronic abdominal pain.
Lichtman et al. (2018) [26]	Adults with advanced cancer pain refractory to opioids. Exclusion criteria included history of schizophrenia, substance abuse, using > 1 opioid, >500 mg morphine equivalents/day.	There are no significant differences between groups on NRS score pain improvement (10.7% improvement vs. 4.5% improvement, *p* = 0.085).	Nabiximols were not superior to placebo as adjunctive therapy for advanced cancer patients.
Narang et al. (2008) [27]	Adults with chronic non-cancer pain (≥4 NRS) refractory to opioids (≥6 months). Exclusion criteria: <8 h between opioids, transdermal/intrathecal opioids, psychiatric disorder, substance abuse, cancer.	Phase I: significant total pain relief with dronabinol (20 mg = 41.7, *p* < 0.01; 10 mg = 39.7, *p* < 0.05). Phase II: significant decrease from baseline average pain scores with dronabinol (*p* < 0.001).	Dronabinol provided adequate analgesia when used as adjunctive therapy for chronic pain.
Notcutt et al. (2004) [28]	Adults with chronic pain. Exclusion criteria included severe comorbidity, psychiatric disorder, substance abuse, history of recreational cannabis use.	Improved S1 VAS scores for THC (*p* < 0.01) and THC: CBD (*p* < 0.05). Improved S2 VAS scores for THC (*p* < 0.001). Improved sleep quality with THC: CBD (*p* < 0.001), THC (*p* < 0.001), and CBD (*p* < 0.05).	THC and THC: CBD extracts greatly improved pain and sleep quality.

**Table 2:** Summary of the use of cannabinoids on neuropathic or pain or pain related to chronic pain diseases such as fibromyalgia or MS.

Author (y)	Groups Studied and Interventions	Results and Findings	Conclusions
Ware et al. (2010) [29]	Adults with post-surgical or post-traumatic neuropathic pain (>4 on VAS) for ≥ 3 months. Requirements: normal liver and renal function, hematocrit > 38%, negative pregnancy test. Exclusion criteria: cancer, severe comorbidity, substance abuse, history of psychosis, suicide, pregnancy, breastfeeding.	VAS pain scores for 9.4% THC were significantly reduced (9.4% THC = 5.4, 0% THC = 6.1, *p* = 0.023); 9.4% THC improved sleep quality and symptoms of anxiety and depression (*p* < 0.05 for both).	Smoked cannabis improved pain, mood, and sleep quality in chronic neuropathic pain.
Weizman et al. (2018) [32]	27–40-year-old men with chronic lumbar radicular pain for > 6 months. Women and patients with other comorbidities were excluded.	THC decreased ACC and sensorimotor cortex functional connectivity in right SII, left SII, and right MI, which correlated with improved pain (r = 0.68, *p* = 0.005; r = 0.66, *p* = 0.007; r = 0.8, *p* = 0.0003, respectively).	THC may reduce subjective neuropathic pain by interfering with neural pathways in the ACC.
van de Donk et al. (2019) [34]	Adult females with fibromyalgia and NRS pain score ≥ 5. Exclusion criteria included neuropsychiatric disorders, use of opioids or benzodiazepines, substance abuse, pregnancy, breastfeeding, recent cannabis use, pain disorder other than fibromyalgia.	No significant differences between groups for NRS pain scores or electrical pain threshold; 30% pain reduction in 18 bediol patients (*p* = 0.01). Bediol and bedrocan enhanced pressure threshold (*p* < 0.001 and *p* = 0.006, respectively).	Bedrocan and bediol reduced pressure threshold, but no group significantly reduced pain NRS scores or electrical pain threshold.
Chaves et al. (2020) [38]	Adults with fibromyalgia with moderate-severe symptoms. Exclusion criteria included comorbidity, psychiatric illness, another disorder causing pain, pregnancy, breastfeeding, cannabis sensitivity.	Significant decline in FIQ scores in the cannabis group (cannabis = 30.50 ± 16.18, placebo = 61.22 + 17.30, *p* = 0.005).	THC oil improved the quality of life in fibromyalgia patients.
Ware et al. (2010) [40]	Adult fibromyalgia patients with chronic insomnia for 6 months. Exclusion criteria: cancer, use of monoamine oxidase inhibitors, neuropsychiatric illness, urinary retention, or sensitivity to study drugs.	Nabilone was superior in sleep quality (difference = −3.25, 95% CI = −5.26 to −1.24). No significant differences on Leeds Sleep Evaluation Questionnaire, but nabilone showed more restful sleep (difference = 0.48, 95% CI = 0.01–0.95) and quicker sleep onset (difference = −0.7, 95% CI = −1.36–0.03).	Nabilone was effective in improving sleep quality in fibromyalgia patients with chronic insomnia.
van Amerongen et al. (2018) [44]	Adults with progressive MS and severe pain and spasticity. Patients were excluded if they had epilepsy, recent disease worsening, or severe cardiac, renal, or hepatic disease.	Improvement from baseline spasticity on Modified Ashworth Scale, 9-Hole Peg Test, and subjective NRS (*p* = 0.001, *p* = 0.018, and *p* = 0.001, respectively). No change in gait or H/M ratio from baseline. Prolonged CSP duration in MS patients (47.9 ± 6.2, *p* = 0.001).	Oral THC:CBD spray was effective in reducing spasticity in MS patients.

**Table 3:** Summary of studies regarding pain and psilocybin.

Author (y)	Groups Studied and Interventions	Results and Findings	Conclusions
Johnson et al. (2012) [53]	18 healthy volunteers were randomly selected to receive either escalating or de-escalating doses of psilocybin (0, 5, 10, 20, 30 mg) over 5 total, 8 h long sessions.	Mean onset of headache was 7.0 h after administration. The number of participants who reported a headache increased as the dose increased, with nearly all reporting headaches at 30mg.	Psilocybin causes headaches in a dose-related fashion.
Ramachandran et al. (2018) [54]	Case report of a patient who had intractable phantom limb pain after amputation described as a nail boring into the leg.	Psilocybin was paired with MVF, where a nail was visualized by the patient being removed from the leg.	Psilocybin-MVF worked synergistically to eliminate the pain that felt and decreased any paroxysmal episodes.

## Data Availability

All data included in this review are available to the the public and is available on PubMed.
